# First person – Conor McGrath

**DOI:** 10.1242/dmm.049579

**Published:** 2022-04-29

## Abstract

First Person is a series of interviews with the first authors of a selection of papers published in Disease Models & Mechanisms, helping early-career researchers promote themselves alongside their papers. Conor McGrath is first author on ‘ Development of a novel human intestinal model to elucidate the effect of anaerobic commensals on *Escherichia coli* infection’, published in DMM. Conor conducted the research described in this article while a PhD researcher in Stephanie Schüller's lab at the University of East Anglia, Norwich, UK. He is now a fermentation technician in the lab of Shane Gaudin at Lysine Australia, Tingalpa, Australia, investigating the harnessing of microbes to benefit human health and biotechnology.

**How would you explain the main findings of your paper to non-scientific family and friends?**

Diarrhoea is a major cause of mortality worldwide, particular amongst infants. Understanding how our native gut bacteria may protect from infection by diarrhoea-causing pathogens may be crucial for effective treatment of the disease. In this study, we aimed to study the protective effects of several gut symbionts on the pathogen enteropathogenic *E. coli*, which has been estimated to cause hundreds of thousands of deaths worldwide. To do this, we first simulated the human intestine by growing intestinal cells using specialized equipment. This re-created several features of the intestine, including low-oxygen levels and the production of mucus on the surface of the cells. We then used this system to demonstrate protective effects of natural gut bacteria on *E. coli* by reducing their ability to inhabit the intestine and cause inflammation. Interestingly, the presence of mucus on the cells was crucial to these protective effects, highlighting its importance to studying human-bacterial interactions.

**Figure:**
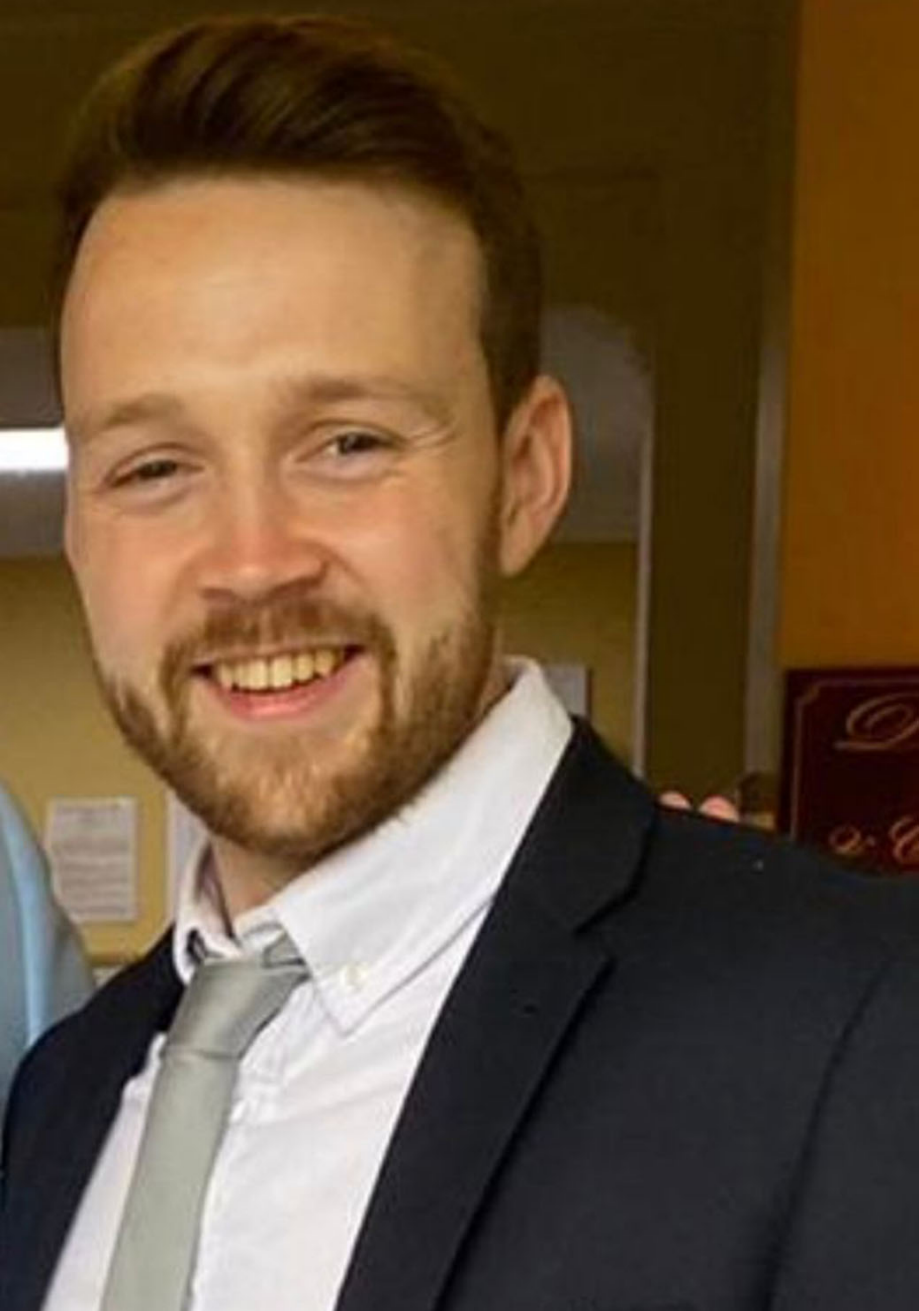
Conor McGrath

**What are the potential implications of these results for your field of research?**

The human gut is dominated by oxygen-sensitive anaerobes, and many pathogens regulate virulence genes in response to oxygen levels. Additionally, the intestinal epithelium is covered by a mucus layer, which bacteria must colonize effectively in order to persist in the host. The results from this work show that anaerobic bacteria can be cultured alongside a human intestinal epithelium, providing a model that can be used to study the multitude of anaerobic bacteria inhabiting the gut. Furthermore, the presence of mucus promotes epithelial adherence of gut bacteria, and allows commensals to protect more robustly from pathogen colonization and downstream inflammation. Including secreted mucus in epithelial models may reveal previously unknown benefits of commensal organisms, emphasising their importance to human health.

**Figure:**
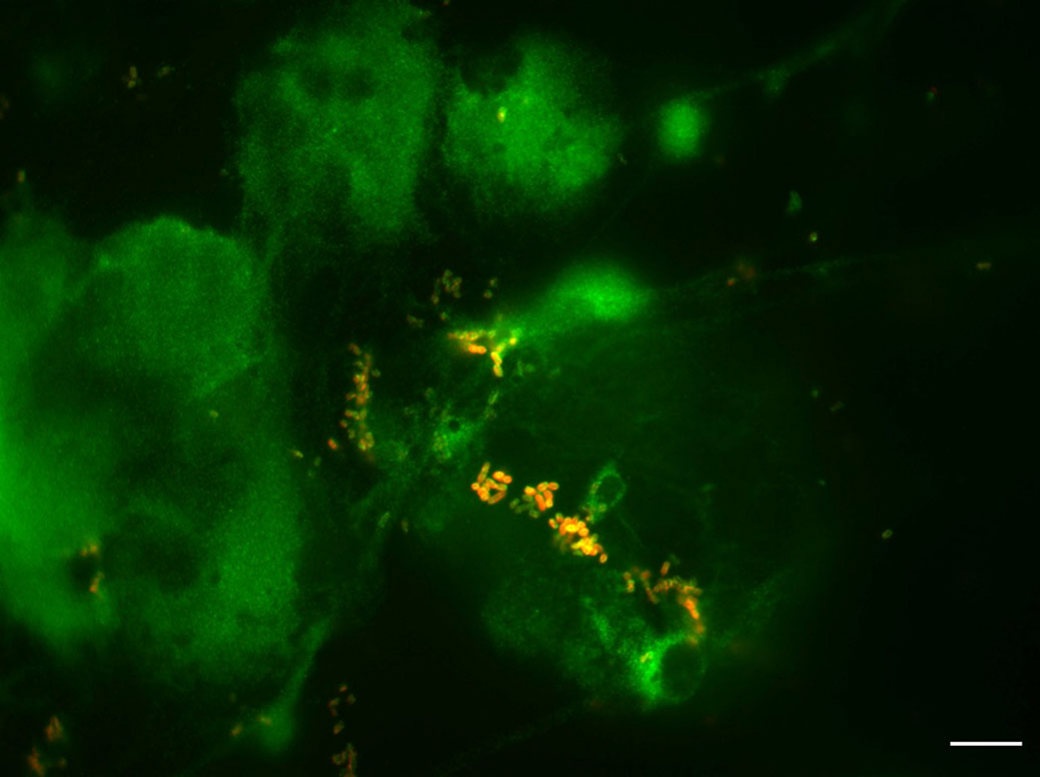
**Interaction of *Limosilactobacillus reuteri* (red) with mucus secreted by the T84/LS174T intestinal epithelium model (green).** Scale bar: 20 µm.

**What are the main advantages and drawbacks of the model system you have used as it relates to the disease you are investigating?**

The diarrhoeagenic pathogen enteropathogenic *E. coli* is highly adapted to its host, hence is unable to recreate disease in commonly used animal models, such as mice. This model employs human-derived intestinal cell lines, which share many features of native tissue and survive in continued culture. The vertical diffusion chamber culturing system used here allows direct access of the bacteria to the host epithelium under microaerobic conditions. These are crucial for expression of the type III secretion system, which injects effector proteins directly in the host cell to disrupt signalling pathways and cause disease.

One major drawback is the reliance on cell lines. Despite their dependability in continued culture, cell lines are derived from carcinomas, which do not fully represent healthy tissue. Additionally, they do not recreate the 3D architecture of the intestinal epithelium, such as villi and crypts. As EPEC is highly adapted to the host, these changes to the suite of colonizable niches may impact translation of the results to natural infection.

**What has surprised you the most while conducting your research?**

What surprised me the most was how sensitive the bacteria were to their environment and the state of the host cells. This is particularly true of the anaerobic bacteria, which require a great deal of preparation and care to culture. At certain points during my PhD project, this involved speed-walking to the neighbouring Quadram Institute, bacteria in tow, to perform downstream tests from VDC experiments. It was fascinating to see the bacteria respond so differently to host cells by changing a single feature, such as polarized versus non-polarized epithelia, as well as with and without goblet cells. Throughout this work, I developed deep appreciation for the finely tuned molecular mechanisms underpinning these interactions, which gently reminded me that our gut bacteria have evolved alongside us for thousands of years.

**Describe what you think is the most significant challenge impacting your research at this time and how will this be addressed over the next 10 years?**

The biggest challenge facing this research and studies into host-microbe interactions in the gut is the development of effective model systems. The model system here is one of many attempts to capture the complexity of the human intestine and tackle some of the biggest health threats facing society. Although a perfect model system is not considered a realistic target owing to the significantly dynamic and diverse nature of the human gut, there are some promising breakthroughs using organoids alongside platforms that mimic physiological conditions, such as low oxygen or peristalsis. Over the next 10 years, these models could be further developed by introducing complex microbiotas and harnessing multi-omics tools to gain incredibly detailed insights into host-microbe interactions. Although it is highly unlikely to be achieved in the next 10 years, the aim of targeted disease interventions using model systems appropriate to the individual remain a realistic goal.

“The model system here is one of many attempts to capture the complexity of the human intestine and tackle some of the biggest health threats facing society.”

**What changes do you think could improve the professional lives of early-career scientists?**

The pressure cooker created by burgeoning post-graduate researchers competing for a limited number of career opportunities has caused many great scientists I know to pursue opportunities in other fields. The lack of post-doctoral research grants and limited research opportunities in industry need to be addressed. Government funding could be funnelled into short-term projects that follow-on from post-graduate research, providing a bridge to the next step career-wise. Additionally, R&D companies should be encouraged to hire graduates through organised schemes that provide clear career progress through the company. Having more opportunities and a more defined career progression would reduce the anxiety and uncertainty around careers in research.

**What's next for you?**

I emigrated to Australia at the end of 2021 to live and work and am currently working for a biotech company looking to generate lysine from bacterial fermentation. Although this is a side-step from host-microbe interactions, I am enjoying being able to use my knowledge of microbiology for commercial applications. Commercialization of research is still my primary focus career-wise; I'm keen to produce technology that could benefit society, be that in the form of human health or environmental impact.
